# The outcomes of 1120 severe multiple trauma patients with hemorrhagic shock in an emergency department: a retrospective study

**DOI:** 10.1186/1471-227X-13-S1-S6

**Published:** 2013-07-04

**Authors:** Yin Wen, Huang Yang, Wang Wei, Liu shan-shou

**Affiliations:** 1 Department of Emergency Xijing Hospital, the Fourth Military Medical University, Changle Xilu Road No.127, Xi'an 710032, China

## Abstract

**Objective:**

Uncontrolled hemorrhagic shock is a significant factor in death of severe multiple trauma patients. The acute management of injured bleeding in emergency department (ED) may improve patient outcomes. The medical records of severe multiple trauma patients with hemorrhagic shock in our ED were reviewed to summarize an evidence-based approach to the management of critically injured bleeding trauma patients.

**Methods:**

A retrospective study was carried out from January 2002 to December 2011 in a Chinese tertiary hospital. Clinical data from major trauma patients with hemorrhagic shock admitted to ED were evaluated. The patients were stratified based on the characteristics of traumatic condition and resuscitation strategies. The medical treatments and the outcomes of these severe multiple trauma patients were described.

**Results:**

A total of 1120 major trauma patients, consisting of 832 males and 288 females, were enrolled. 906 of the patients (80.9%) were injured in traffic accidents, 104 (9.3%) from falling, and 100 from other reasons. The number of injured sites varied from 2 to 6, 616(55.0%) more than 3. 902 (80.5%) trauma patients have recovered and been discharged from hospital.

**Conclusions:**

Uncontrolled hemorrhagic shock is a main reason of trauma patients’ death. The resuscitation strategy should center upon permissive hypotension and early hemostatic resuscitation combined identified and corrects coagulopathy. The current approach to the management of critically injured bleeding trauma patients is able to improve patient outcomes.

## Introduction

The care of major trauma patients continues to be a challenge for emergency physicians and trauma surgeons. Uncontrolled hemorrhagic shock is a significant factor in death of severe multiple trauma patients.[1] It is estimated that acute management of injured bleeding in emergency department (ED) could improve patient outcomes.[2] Doctors also believe that resuscitation strategies should be carried out as soon as possible.[3] The present study was undertaken to analyze the medical records of severe multiple trauma patients with hemorrhagic shock, with the objective of providing a scientific basis for an evidence-based approach to the management of critically injured bleeding trauma patients.

## Methods

### Study design

This retrospective study was carried out in a Chinese tertiary general hospital with 3500 inpatient beds, which works as a level I trauma center. Ethical approval was not required. All severe trauma patients with hemorrhagic shock who were admitted to ED were enrolled.

### Medical treatments

Upon the patients arriving in ED, emergency doctors rapidly make a preliminary assessment of the traumatic conditions through observing consciousness level and respiratory rhythm, monitoring heart rate and blood pressure, examining chest, abdomen and limb activity. Making a brief and accurate examination to find out and treat immediately the life-threatening injuries, such as respiratory obstruction, tension pneumothorax, bleeding and hypotension. Control active bleeding of wound on body surface by pressure bandage-fixing therapy.

If sustained hypotension existing, doctors will determine the shock degree, estimate blood loss volume, and give anti-shock fluid resuscitation. The common treatments in our ED: Quickly open two vein channels, make a deep vein catheterization if necessary. Infusion rapid of 1000-1500ml balanced salt solution and 500-1000ml 706 plasma substitutes or Voluven in the first 20-30minutes. Give coagulation support and monitoring, such as transfusing packed red blood cells, fresh-frozen plasma, platelet, cryoprecipitate, rFVII2 and tranexamic acid to correct coagulopathy. The aim is to maintain patients’ blood pressure around (90-80)/(60-50) mmHg before bleeding was controlled. Perfect preoperative examination and start damage control surgery within 1 hour if necessary.

## Results

From January 2002 to December 2011, a total of 1120 major trauma patients, consisting of 832 males and 288 females, were enrolled. 906 of the patients (80.9%) were injured in traffic accidents, 104 (9.3%) from falling, and 100 from other reasons. The number of injured sites varied from 2 to 6, 616(55.0%) more than 3. The most common injured site was the head (822 patients, 73.4%), followed by the extremities and pelvis (626 patients, 55.9%), the chest (480 patients, 42.9%) and the abdomen (384 patients, 34.3%).

94 (8.4%) patients died in the rescue room before been transported to emergency intensive care unit (EICU) , 124(11.1%) died or withdrawal of therapy by the family due to medical expenses and other reasons in EICU. 902 (80.5%) trauma patients recovered, and were discharged from hospital.

## Discussion

Uncontrolled hemorrhagic shock after injury continues to be a challenge for emergency physicians, which is a main reason of death of trauma patients within the first 2-3 hours after the injury. It was reported that 20% multiple trauma patients died in this period.[1]

A multidisciplinary task force for advanced bleeding care in trauma was formed in 2005 with the aim of developing a guideline for the management of bleeding following severe injury. This group published the first and an updated version of guideline in 2007 and 2010 respectively, which provides an evidence-based multidisciplinary approach to the management of critically injured bleeding trauma patients based on a systematic review of published literature. The newest 2010 guideline[1]include new recommendations on coagulation support and monitoring and the appropriate use of local haemostatic measures, tourniquets, calcium and desmopressin in the bleeding trauma patient. Holeomb reported that identified and corrects coagulopathy can increase rescue success rate.[4] In our study, we also found that the patients with coagulopathy were more likely to die.

Different to traditional management of injured bleeding trauma patients which centered upon correction of acidosis and hypotension with crystalloids. Damage control resuscitation (DCR), a new resuscitation strategy, is permissive hypotension and early hemostatic resuscitation combined identified and corrects coagulopathy with fresh-frozen plasma (FFP), restricting use of crystalloids.[5,6] In our hospital, doctors now maintain patients’ blood pressure around (90-80)/(60-50) mmHg before bleeding was controlled. The trauma patients who received blood transfusion, such as packed red blood cells, fresh-frozen plasma, platelet, cryoprecipitate, rFVII2 and tranexamic acid, seemed have a better outcome. 80.5% trauma patients recovered, which is a great deal higher than before.

## Conclusions

In conclusion, immediately find out and treat the life-threatening bleeding and hypotension, identify and correct coagulopathy, damage control resuscitation are helpful to manage critically injured bleeding trauma patients. In order to improve patient outcomes, this evidence-based approach is worthy of further practice and popularization.

## Declarations

This article has been published as part of *BMC Emergency Medicine* Volume 13 Supplement 1, 2013: Proceedings of the 2012 Emergency Medicine Annual Congress. The full contents of the supplement are available online at http://www.biomedcentral.com/bmcemergmed/supplements/13/S1.

The publication costs for this article was funded by Xijing Hospital, the Fourth Military Medical University.
